# Regulation of Angiogenesis by Non-Coding RNAs in Cancer

**DOI:** 10.3390/biom14010060

**Published:** 2024-01-02

**Authors:** Zhiyue Su, Wenshu Li, Zhe Lei, Lin Hu, Shengjie Wang, Lingchuan Guo

**Affiliations:** 1 Department of Pathology, The First Affiliated Hospital of Soochow University, Suzhou 215006, China; 2 State Key Laboratory of Radiation Medicine and Protection, School of Radiation Medicine and Protection, School for Radiological and Interdisciplinary Sciences (RAD-X), Collaborative Innovation Center of Radiation Medicine of Jiangsu Higher Education Institutions, Soochow University, Suzhou 215123, China; 3 Department of Basic Medicine, Kangda College, Nanjing Medical University, Lianyungang 222000, China

**Keywords:** miRNA, lncRNA, circRNA, tumor angiogenesis

## Abstract

Non-coding RNAs, including microRNAs, long non-coding RNAs, and circular RNAs, have been identified as crucial regulators of various biological processes through epigenetic regulation, transcriptional regulation, and post-transcriptional regulation. Growing evidence suggests that dysregulation and activation of non-coding RNAs are closely associated with tumor angiogenesis, a process essential for tumor growth and metastasis and a major contributor to cancer-related mortality. Therefore, understanding the molecular mechanisms underlying tumor angiogenesis is of utmost importance. Numerous studies have documented the involvement of different types of non-coding RNAs in the regulation of angiogenesis. This review provides an overview of how non-coding RNAs regulate tumor angiogenesis. Additionally, we discuss emerging strategies that exploit non-coding RNAs for anti-angiogenic therapy in cancer treatment. Ultimately, this review underscores the crucial role played by non-coding RNAs in tumor angiogenesis and highlights their potential as therapeutic targets for anti-angiogenic interventions against cancer.

## 1. Introduction

Non-coding RNA (ncRNA) refers to a class of RNA molecules that are transcribed from the genome but do not have the ability to code for proteins [1,2]. They can be classified into two main types: housekeeping ncRNA and regulatory ncRNA [3]. Regulatory ncRNAs can be further categorized as microRNA (miRNA), small interfering RNA (siRNA), piwi-interacting RNA (piRNA), long non-coding RNA (lncRNA), and circular RNA (circRNA) [3]. In recent years, there has been extensive research on regulatory ncRNAs, especially in the field of cancer research. These regulatory ncRNAs play important biological functions through epigenetic regulation, transcriptional regulation, and post-transcriptional regulation.

Tumor angiogenesis refers to the process of new blood vessel formation during the development of malignant tumors, which provides nutrients and oxygen to tumor cells [4,5]. Moreover, tumor angiogenesis offers a pathway for tumor metastasis, which is the leading cause of death in cancer patients [6,7]. Sustaining angiogenesis is a significant hallmark of cancer [8]. When vascular support is lacking, tumors may become necrotic or even apoptotic [9]. Therefore, targeting angiogenesis is a promising strategy for cancer treatment [10].

In recent years, there has been growing evidence suggesting that miRNA, lncRNA, and circRNA play a significant role in the regulation of tumor angiogenesis. It has become necessary to categorize and summarize the molecular mechanisms of various ncRNAs in tumor angiogenesis. This article aims to summarize the functions of various ncRNAs during tumor angiogenesis and discuss their potential implications for tumor diagnosis and treatment.

## 2. The Characteristics of Tumor Angiogenesis

Angiogenesis is a sequential, multi-step process that includes the destruction of the extracellular matrix, the budding and elongation of endothelial cells, the migration and proliferation of endothelial cells, and the formation and maturation of tubes [11]. A variety of cell types, including tumor cells, endothelial cells, immune cells, and fibroblasts are involved in tumor angiogenesis, which correlates with the complexity of the tumor microenvironment (TME) [12]. There is a diverse group of mediators secreted from these cells including growth factors, matrix-degrading enzymes, cytokines, bioactive lipids, and a variety of small molecules in TEM [13]. Among these mediators, vascular endothelial growth (VEGF) factors are thought to play a crucial role in regulating tumor angiogenesis. It can activate intracellular signaling pathways by binding to the corresponding receptors on endothelial cell membranes, ultimately forming blood vessels [14]. Hypoxia is a major feature of the tumor microenvironment, leading to the activation of the hypoxia-inducible factor-1 (HIF-1) transcription factor in tumor cells, which promotes the expression of VEGF [15,16]. HIF-1 complex activity can also be influenced by inflammation and cellular stress among other factors [17]. Fibroblasts accumulate in the early stages of tumor tissue formation and participate in regulating angiogenesis by secreting plasminogen activators (PAs) [18]. Immune cells, such as tumor-associated macrophages, secrete various growth factors and chemical mediators like VEGF, fibroblast growth factor-2, and angiogenesis-modulating enzymes. These substances directly or indirectly affect the process of angiogenesis [19]. In addition, several signaling pathways, such as transforming growth factor-beta (TGF-β) and signal transducer and activator of transcription (STAT), are involved in regulating the expression of these mediators [20,21]. The above features contribute to the complex nature of tumor angiogenesis. Additionally, these newly formed blood vessels often exhibit irregular, incomplete, and highly permeable characteristics, which promote cancer cell growth and metastasis [5,22]. Taken together, tumor angiogenesis is a complex process characterized by multi-step composition, the involvement of multiple cell types, and multi-factorial regulation.

## 3. miRNA and Tumor Angiogenesis

**Figure 1:**
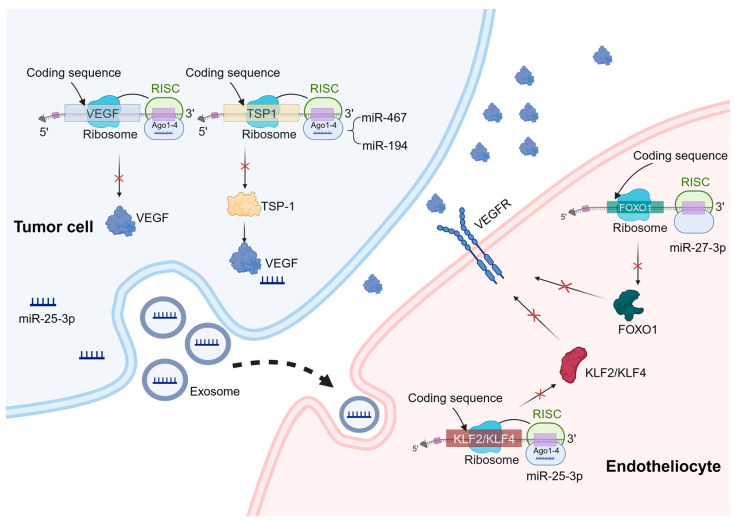
Mechanisms of miRNA regulation of tumor angiogenesis. miRNAs can play a role in tumor cells and vascular endothelial cells to regulate tumor angiogenesis. In the process of tumor angiogenesis, miRNAs primarily function by binding to the 3′-UTR of specific mRNAs, leading to mRNA degradation or translation inhibition. These target genes mainly include pro-angiogenic or anti-angiogenic factors, as well as genes involved in angiogenesis signaling pathways. A similar mechanism also exists in vascular endothelial cells. For example, miR-27b-3p targets the 3′-UTR region of FOXO1 mRNA and downregulates its expression, thereby regulating the expression of VEGFR. In addition, exosomal miRNAs have a significant effect on angiogenesis. For example, miR-25-3p targets and silences KLF2 and KLF4, ultimately regulating the expression of genes associated with VEGFR2 and ZO-1 in endothelial cells.

miRNAs are a type of small RNA molecules that exist naturally within organisms and are approximately 20–24 nucleotides long [23]. They are present in various organisms and play a significant role in regulating gene expression [24]. They achieve this regulation mainly by targeting specific recognition sites in the 3′ untranslated region (UTR), leading to mRNA degradation [25] (Figure 1). Dysregulation of miRNAs can lead to the development and progression of several diseases. In particular, aberrant miRNA expression has been linked to tumor angiogenesis [26,27]. Understanding the roles and behaviors of miRNAs offers valuable insights into the mechanisms of angiogenesis and potential targets for therapeutic interventions. The role of miRNAs in tumor angiogenesis is complex and multifaceted. We have classified the recent studies in Table 1 to highlight the functions of miRNAs in the following aspects.

miRNAs have a promoting effect on tumor angiogenesis. Since miRNAs are repressive for the regulation of target genes, angiogenesis inhibitors such as platelet response protein 1 (TSP-1) become prime targets when miRNAs promote tumor angiogenesis [50]. It has been demonstrated that miR-467 targets TSP-1, leading to increased inflammation resolution and angiogenesis [51]. Moreover, overexpression of miR-194 has been observed in advanced colorectal cancer, which binds to the 3′-UTR region of the TSP1 gene [52]. On the other hand, when miRNAs directly regulate angiogenic factors, they inhibit tumor angiogenesis. One crucial group of angiogenic factors is the VEGF family, and overactivation of these factors can result in abnormal angiogenesis [53]. For example, studies have demonstrated that miR-126 downregulates VEGF-A expression, inducing apoptosis and impeding tumor angiogenesis across various cancer types including breast cancer, lung cancer, oral cancer, and esophageal cancer [54,55,56,57].

miRNAs also play important roles in vascular endothelial cells to regulate tumor angiogenesis. Specifically, miR-27b can suppress the activation of inflammatory pathways, consequently inhibiting intrinsic apoptosis. Mechanistically, miR-27b-3p targets the 3′-UTR region of FOXO1 mRNA, thereby downregulating its expression and subsequently attenuating the activation of the AKT/FOXO1 pathway [58].

In addition to miRNAs present within endothelial cells, extracellular vesicle miRNAs (exosomal miRNAs) have a notable impact on angiogenesis. For instance, recent studies have revealed that exosomal transfer of miR-25-3p from colorectal cancer (CRC) cells to endothelial cells promotes CRC metastasis. Mechanistically, this exosomal miRNA targets and silences KLF2 and KLF4, ultimately regulating gene expression associated with VEGFR2 and ZO-1 within endothelial cells. Consequently, this process promotes vascular permeability and neovascularization [59]. Importantly, extracellular vesicle miRNAs can also exert their influence on tumor angiogenesis by modulating immune cells. Research conducted by Zhao et al. demonstrates that exosomal miR-934 derived from CRC cells induces M2 macrophage polarization through downregulation of PTEN expression and activation of the PI3K/AKT signaling pathway. As a result, M2 macrophages produce various growth factors and cytokines like CXCL13 and CXCR5 that play crucial roles in regulating tumor growth, facilitating migration, and promoting angiogenesis [60]. In vivo, exosomes derived from stem cells of human deciduous exfoliated teeth significantly reduce the micro-vascular formation of tumors generated from xenografted oral squamous cell carcinoma cells via the transfer of miR-100-5p and miR-1246 [61].

## 4. lncRNA and Tumor Angiogenesis

**Figure 2:**
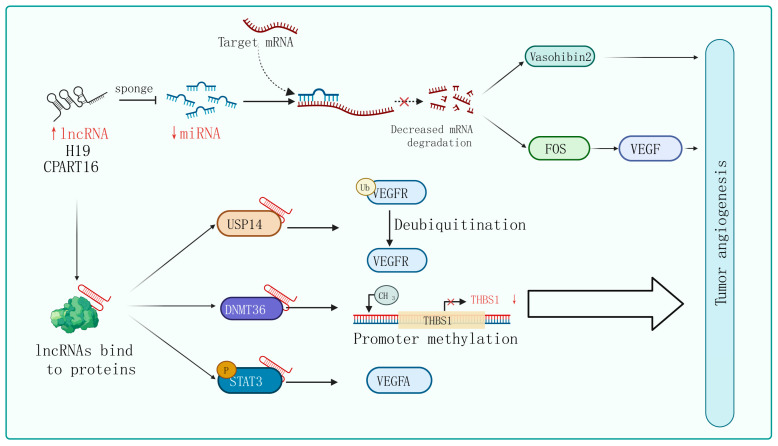
Mechanisms of lncRNA regulation of tumor angiogenesis. In certain tumor cells and specific tissues, some lncRNAs carry specific sequences that can adsorb miRNA, acting in a similar way to sponges in order to bind with miRNA, thereby preventing miRNA from binding to its target mRNA. On the other hand, lncRNA can interact with certain proteins, affecting their post-translational modifications, protein stability, transcription, and translation activities, ultimately affecting tumor angiogenesis through the regulation of downstream target genes.

lncRNAs, a group of RNA molecules longer than 200 nucleotides with a structure similar to mRNA, have been shown to possess more versatile mechanisms for regulating gene expression compared to miRNAs [62,63,64]. In recent years, lncRNAs have been implicated in various biological processes, including tumor proliferation, migration, invasion, and angiogenesis [65]. Here, we aimed to present an overview of the molecular mechanisms by which lncRNAs function as miRNA sponges, protein scaffolds, and coding peptides in the context of tumor angiogenesis (Figure 2). Table 2 categorizes recent studies on lncRNAs’ involvement in tumor angiogenesis.

The phenomenon of lncRNAs acting as sponges that absorb miRNAs to promote target gene expression is universal in tumor angiogenesis. For instance, lncRNA H19 is upregulated in glioblastoma and plays an important role in driving angiogenesis by sequestering miR-29a and increasing the expression of vasohibin 2, an angiogenesis factor [87]. Similarly, Zhang et al. identified an lncRNA called CRART16 that is significantly overexpressed in gastric cancer tissue. They found that CRART16 acts as a sponge for miR-122-5p and upregulates the expression of the oncogene FOS. The overexpression of FOS leads to an increase in VEGF levels, promoting cancer cell growth and angiogenesis [73]. In addition, using the xenograft animal model, lncRNA ZNRD1-AS1 has been proven to inhibit the development of lung cancer by attenuating tumor angiogenesis [66].

The interactions between lncRNAs and various types of proteins confer a diversity of modalities for regulating tumor angiogenesis. Firstly, they can influence post-translational modifications of proteins. For instance, lncRNA PCAT6 binds to USP14 (a deubiquitinase) to induce the deubiquitination of VEGFR2, thereby increasing VEGFR2 expression levels and promoting angiogenesis in triple-negative breast cancer (TNBC) [68]. Secondly, lncRNAs can regulate gene transcription and translation. For example, in breast cancer cells, lncRNA RAB11B-AS1 promotes VEGFA and ANGPTL4 gene transcription by recruiting RNA Pol II to their promoter, enhancing tumor angiogenesis [82]. Additionally, lncRNA BZRAP1-AS1 indirectly enhances the methylation of the THBS1 promoter by increasing the stability of the DNMT3b protein, which inhibits the transcription of the anti-angiogenic gene THBS1 and promotes the angiogenesis process in tumors [88]. Furthermore, lncRNA HITT weakens the binding between YB-1 proteins and the 5′-UTR of HIF1α mRNA, impairing HIF1α translation. Finally, lncRNA can regulate signaling pathway activity. lncPVT1 interacts with phosphorylated STAT3 directly in the cell nucleus and activates the STAT3 signaling pathway, leading to increased expression of VEGFA [89]. Similarly, in non-small-cell lung cancer, researchers have found that lncRNA EPIC1 promotes tumor angiogenesis by activating the Ang2/Tie2 axis [90].

Although lncRNAs do not have typical protein-coding ability, some specific lncRNAs are capable of encoding small peptides due to their open reading frames (ORFs). Researchers found that LINC00908 can encode a 60-amino acid peptide in the TNBC. Interestingly, the peptide can directly interact with STAT3 and reduce the phosphorylation level of STAT3, thereby decreasing the expression of VEGF [91].

## 5. cirRNA and Tumor Angiogenesis

**Figure 3:**
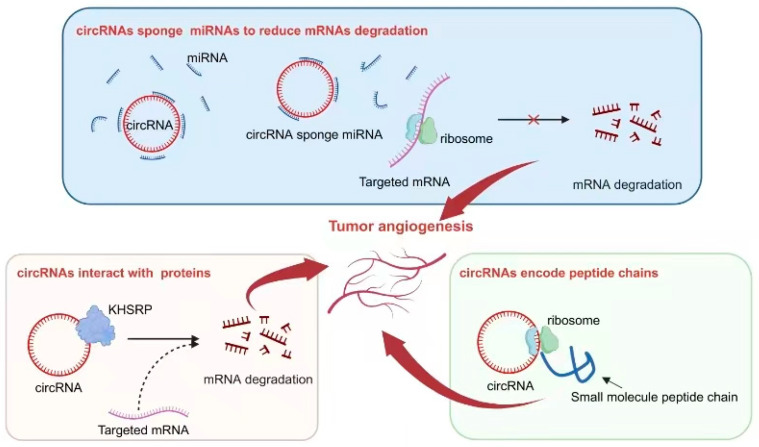
Mechanisms of circRNA regulation of tumor angiogenesis. Some circRNAs function as sponges, absorbing miRNAs, inhibiting the binding of miRNAs to target genes, reducing the degradation of target mRNA, and thereby affecting tumor angiogenesis. Certain circRNAs have binding sites with the protein KHSRP, influencing the degradation of target mRNA and impacting tumor angiogenesis. The small peptides encoded by circRNAs play a role in influencing tumor angiogenesis.

circRNAs are a unique form of non-coding RNA that possesses a closed circular structure, providing it with exceptional stability. Consequently, circRNAs are abundantly found in various cells and tissues and play significant roles in diverse biological processes, including tumor angiogenesis. Previous studies have elucidated several mechanisms by which circRNAs function: (1) They act as sponges, sequestering miRNAs and thereby inhibiting their regulatory effects on target genes. (2) They interact with proteins to modulate their localization, post-translational modifications, and stability. (3) Notably, circRNAs themselves can serve as potential sources for encoding small functional peptides that exhibit specific biological activities (Figure 3).

As a crucial regulator of tumor angiogenesis, VEGF serves as an excellent indicator for investigating the role of circRNAs in regulating this process. The abnormal activation of circRNAs often directly or indirectly influences the expression of VEGF, thereby exerting an impact on tumor angiogenesis. For instance, circ-29 enhances gastric cancer invasion and angiogenesis by sequestering miR-29a and consequently boosting the VEGF signaling pathway. The underlying mechanism involves the interaction between circ-29 and miR-29a, which reduces the latter’s ability to target the 3′-UTR region of VEGF mRNA and ultimately increases VEGF expression. Elevated levels of circ-29 contribute to the enhancement of invasive capabilities in gastric cancer cells and the promotion of neovascularization within the tumor microenvironment [92]. Furthermore, we have compiled and summarized the recent studies exploring how circRNA–miRNA interactions regulate various types of tumor angiogenesis in Table 3.

Interactions between circRNAs and various proteins play a crucial role in the process of tumor angiogenesis. One example is circLMP2A, which forms a complex with KHSRP, an RNA-splicing regulatory protein. This interaction inhibits the stability of VHL mRNA and relieves its inhibitory effect on HIF1α-induced VEGFA expression [93]. Another notable interaction involves circSHKBP1 and HSP90. In this case, their interaction inhibits the degradation of HSP90 by STUB1, the E3 ubiquitin ligase, resulting in enhanced VEGF expression in tumor tissue. As a result, progression and angiogenesis are enhanced in gastric cancer [94]. Table 3 provides further classification and documentation for similar mechanisms that have been reported.

Interestingly, some specific circRNAs have been found to possess the capability of encoding small peptides. This ability is attributed to the presence of an ORF within their sequence. In the context of tumor angiogenesis, this phenomenon has been extensively studied and reported. One such example is circ-0000437, which has been identified as encoding a small peptide comprising 47 amino acids. Through research efforts, it has been discovered that this particular peptide plays a significant role in inhibiting the interaction between ARNT and TACC3 proteins. Consequently, this inhibition leads to a reduction in VEGF expression, ultimately resulting in the suppression of tumor angiogenesis [95].

In addition to its effects on tumor cells, circRNA can regulate other cells in the tumor microenvironment through the secretion of exosomes by tumor cells. Exosomes are nanoscale lipid-enclosed structures with a diameter of 30–100 nm that are released by cells and contain proteins, nucleic acids, and other substances. This regulation contributes to the promotion of tumor angiogenesis. For instance, circHIPK3, which is highly expressed in breast cancer, can be carried by exosomes and enter human endothelial cells after being released into the extracellular space. circHIPK3 relieves the inhibitory effect of miR-124-3p on MTDH gene expression, ultimately promoting angiogenesis [96]. Furthermore, other cells from the tumor microenvironment can impact angiogenesis. Studies have demonstrated that M2 macrophages promote angiogenesis in cutaneous squamous cell carcinoma (cSCC). One potential mechanism involves the interaction between circ_TNFRSF21 and miR-3619-5p, leading to the increased expression of the ROCK2 gene and subsequent promotion of angiogenesis [97].

**Table 3:** Targets and dysregulation of circRNA associated with tumor angiogenesis.

Basic Mechanisms	CircRNA	Molecular Target	Cancer Types	The Role in Tumor Angiogenesis	References
Sponging miRNAs (circRNA-miRNA-targeted gene)	CircRNA ARF1	miR-342–3p/ISL2	glioma cancer	promoter	[98]
	Circ_0008344	miR-638/SZRD1	glioma cancer	promoter	[99]
	Circ-ATXN1	miR-526b-3p/VEGFA	glioma cancer	promoter	[100]
	Circ3823	miR-30c-5p/TCF7	colorectal cancer	promoter	[101]
	CircTUBGCP4	miR-146b-3p/PDK2	colorectal cancer	promoter	[102]
	Circ_0030998	miR-567/VEGFA	colorectal cancer	promoter	[103]
	Circ-ZNF609	miR-145/STMN1	nasopharyngeal carcinoma	promoter	[104]
	CircFIRRE	miR-486-3p and miR-1225-5p/LUZP1	osteosarcoma	promoter	[105]
	CircFOXP1	microRNA -127-5p/CDKN2AIP	osteosarcoma	promoter	[106]
	Circ_001587	microRNA-223	pancreatic cancer	inhibitor	[107]
	Circ_000684	miR-145/KLF5	pancreatic ductal adenocarcinoma cells	inhibitor	[108]
	CircRNF13	miR-654-3p/PDK3	pancreatic cancer	promoter	[109]
	CircKDM4B	miR-675/NEDD4L	breast cancer	inhibitor	[110]
	CircHIPK3	miR-124-3p/MTDH	Breast cancer	promoter	[96]
	Circ_0001667	miR-6838-5p/CXCL10	breast cancer	promoter	[111]
	Circ29	miR-29a/VEGF	Gastric cancer	promoter	[92]
	Circ_0001190	miR-586/SOSTDC1	Gastric Cancer	inhibitor	[112]
	Circ_0025033	miR-370-3p/SLC1A5	ovarian cancer	promoter	[113]
	CircNFIX	miR-518a-3p/TRIM44	ovarian cancer	promoter	[114]
	CircATRNL1	miR-378/SMAD4	ovarian cancer	inhibitor	[115]
	Circ_0111738	miR-1233-3p/HIF-1	Multiple Myeloma	inhibitor	[116]
	Circ_0058058	miR-338-3p/ATG14	multiple myeloma	promoter	[117]
	Circ_TNFRSF21	miR-3619-5p/ROCK	cutaneous squamous cell carcinoma	promoter	[97]
	Circfip1L1	miR-125a-5p/VEGFA	Nasopharyngeal Carcinoma Cells	inhibitor	[118]
	CircHIPK2	miR-1249-3p/VEGFA	non-small cell lung cancer	promoter	[119]
	Circ_0006988	miR-491-5p/MAP3K3	non-small cell lung cancer	promoter	[120]
	Circ-RAD23B	miR-142-3p/MAP4K3	non-small cell lung cancer	promoter	[121]
	CircPRRC2A	miR-514a-5p and miR-6776-5p/TRPM3	renal cell carcinoma	promoter	[122]
	CircAFAP1	miR-374b-3p/VEGFA	renal cell carcinoma	promoter	[123]
	Circ_0015004	miR-130a-3p/CEP55	renal cell carcinoma	promoter	[117]
	Circ_0001955	miR-646/FZD4	Hepatocellular Carcinoma	promoter	[124]
	Circ_0000519	miR-1296/E2F7	hepatocellular carcinoma	promoter	[125]
	CircHDAC1_004	miR-361-3p/NACC1	Hepatocellular Carcinoma	promoter	[126]
	Circ_0000144	miR-1178-3p/YWHAH	papillary thyroid cancer	promoter	[127]
	Circ_0011058	miR-335-5p/YAP1	papillary thyroid cancer	promoter	[128]
	CircSHKBP1	miR-766-5p/HMGA2	laryngeal squamous cell carcinoma	promoter	[129]
	Circ_0062019	miR-1253/NRBP1	prostate cancer	promoter	[130]
	CircSLC8A1	miR-21	prostate cancer	inhibitor	[131]
	Circ_0008726	miR-206/HOXA13	Esophageal squamous cell carcinoma	promoter	[132]
Interacting (circRNA-Proteins)	circLMP2A	KHSRP	gastric carcinoma	promoter	[93]
	circKIF18A	FOXC2	Glioblastoma	promoter	[133]
	circSHKBP1	HSP90	gastric cancer	promoter	[94]
	circPOLR2A	UBE3C and PEBP1	clear cell renal cell carcinoma	promoter	[134]
	circFNDC3B	FUS	Oral Squamous Cell Carcinoma	promoter	[135]
	circ_0004018	FUS	hepatocellular carcinoma	inhibitor	[136]
	CircSMARCA5	SRSF1	Glioblastoma	promoter	[137]

## 6. Other Types of ncRNA and Tumor Angiogenesis

Compared to miRNA, lncRNA, and circRNA, there have been fewer research reports on tsRNA, snoRNA, and piRNA in tumor angiogenesis in recent years. Here, we present a classification summary of these molecules. tRNA-derived small RNAs (tsRNAs) are a class of small non-coding RNAs derived from different regions of tRNA, including the 5′ end, 3′ end, and internal regions. tsRNAs can regulate gene expression and thereby influence important biological processes such as cell proliferation, differentiation, and apoptosis [138]. One study has demonstrated that tsRNA-26576 is highly expressed in breast tissues and functions in promoting tumor cell proliferation and inhibiting tumor cell apoptosis [139]. Additionally, 5′tiRNA-His-GTG has been found to be highly expressed in colorectal cancer tissues and plays a role in response to hypoxic stress by activating the HIF1α/vascular axis and promoting the Hippo pathway [140].

Small nucleolar RNAs (snoRNAs) are a diverse class of non-coding RNAs (ncRNAs) that range in length from approximately 60 to 300 nucleotides. These specific ncRNAs are primarily localized in the nucleolus and play important roles in cleaving and chemically modifying ribosomal RNAs (rRNAs) [141,142]. In recent years, there have been limited studies investigating the association between snoRNA and tumor angiogenesis. However, reports have suggested abnormal expression of snoRNAs in various types of tumors, indicating their potential correlation with tumorigenesis. For instance, SNORA42 has been demonstrated to act as an oncogene in lung cancer, hepatocellular carcinoma, and colorectal cancer [143,144,145]. Similarly, upregulation of SNORD17 has been observed in hepatocellular carcinoma tissues compared to normal liver tissues, implicated in driving cancer progression. Mechanistically, SNORD17 anchors nucleophosmin 1 (NPM1) and MYB binding protein 1a (MYBBP1A) simultaneously within the nucleolus, leading to decreased activation of p53 [146].

piRNA is a recently discovered class of small non-coding RNA that is found in both germline cells and somatic cells. These RNA molecules can form complexes with PIWI proteins and typically comprise 24-31 nucleotides in length [147]. In contrast to other RNA sequences, piRNAs deviate from canonical sequences by having a uridine at the 5′ end or an adenine at position ten. Additionally, they lack clear secondary structure motifs [148]. The function of piRNAs encompasses various biological processes such as transposon silencing, spermatogenesis, genome rearrangements, epigenetic regulation, and protein regulation [147]. However, recent reports suggest that abnormal expression of piRNAs may be associated with tumor development and angiogenesis [149]. For instance, studies have observed the upregulation of piRNA-823 in patients with multiple myeloma (MM) as well as MM cell lines. This upregulation positively correlates with clinical staging. In MM cells specifically, downregulating piRNA-823 has been shown to inhibit the secretion of VEGF, subsequently reducing angiogenic activity [150]. Furthermore, low expression levels of piRNA-2158 have been detected in breast cancer tumors. It functions by suppressing transcription through binding to the IL-11 promoter. Interestingly enough, it has been demonstrated that this specific piRNA inhibits angiogenesis in breast cancer, as well [151].

## 7. ncRNA-Targeting Therapeutics in Tumor Angiogenesis

Targeted ncRNA therapy holds great promise for the treatment of tumors. In recent decades, significant clinical investment has been made in RNA-based therapeutic modalities. Currently, there are various RNA-based therapeutic modalities available, such as antisense oligonucleotides (ASOs), small interfering RNAs (siRNAs), short hairpin RNAs (shRNAs), ASO anti-microRNAs (antimiRs), miRNA mimics, miRNA sponges, therapeutic circular RNAs (circRNAs), and CRISPR-Cas9-based gene editing. Among these modalities, ASOs and siRNAs are more widely used [152].

There are several advantages to using miRNA-based therapies. Firstly, miRNAs are naturally occurring molecules in human cells, in contrast to man-made chemotherapeutic compounds or ASOs. They possess all the mechanisms for processing and downstream target selection. Secondly, miRNAs act by targeting multiple genes in a pathway, thereby eliciting a broader but specific response [153,154,155].

One notable characteristic of circRNAs is their closed covalent structure, which makes them less susceptible to degradation compared to other RNA molecules. This property allows circRNAs to easily accumulate within various types of cells or tissues and serve as drugs [156]. Understanding the molecular mechanisms by which circRNAs function has led to the utilization of various chemical and enzymatic synthesis strategies for producing circRNA drugs in vitro [157]. In contrast to conventional protein- or peptide-based drugs, circRNAs exhibit a sub-stoichiometric mode of action, thus facilitating drug delivery with lower toxicity concerns. Moreover, they have favorable pharmacokinetic potential compared to traditional vaccines based on pathogens and DNA/proteins/peptides since they do not require nucleotide modification; yet, they can achieve strong drug efficacy through simple steps [158].

Based on the advantages of targeted ncRNA therapy and the understanding of the molecular mechanism of ncRNA, the first clinical trial of miRNA therapy for tumors was conducted in 2013 [159]. The drug MRX34 is a synthetic double-stranded mir-34a mimic, which can induce tumor cell apoptosis, inhibit tumor cell survival, and extend the survival time of mice. However, severe immune-mediated adverse events occurred in patients in the phase I clinical trial, leading to trial failure [160]. Nevertheless, this experiment proved that improving tumor-specific delivery systems can reduce the off-target toxicity of miRNA drugs. Subsequently, a synthetic mimic of miR-16 called TargomiR entered clinical trials but showed insignificant effects and mild adverse events [161]. However, this clinical trial indicated that the drug delivery system has a greater responsibility for inflammation toxicity. With continuous improvement in drug delivery systems and other conditions, an analog of mir-193a-3p called INT-IB3 is currently undergoing phase I clinical trials [162]. In addition, as of the date of this paper, no circRNA therapeutic candidate has entered clinical trials. However, there has been some progress in pre-clinical studies utilizing circRNA-based mechanisms and nanoparticle delivery systems for cancer treatment. For instance, researchers have discovered that circEHMT1 can effectively inhibit the migration and invasion of breast cancer cells. In a study conducted on mice, nanoparticles were employed to deliver a plasmid expressing circEHMT1, resulting in a significant reduction in lung metastasis of breast cancer cells [163]. Although ncRNA-based therapeutics are still mainly in the research and development stage, it is believed that they will have a wide range of applications in the near future.

## 8. Conclusions and Future Perspectives

This review mainly summarizes the functions of miRNAs, lncRNAs, and circRNAs in tumor angiogenesis and highlights their molecular mechanisms. Meanwhile, we have summarized the advantages of ncRNA-based therapy. It is well understood for the significant roles of miRNAs, lncRNAs, and cirRNAs in tumor angiogenesis.

We have conducted a classification analysis of miRNA, lncRNA, and circRNA based on their molecular mechanisms in tumor angiogenesis. It can be observed that they are interconnected and compete with each other. For example, lncRNAs and circRNAs can inhibit the function of miRNAs by sequestering them. With regard to the summarized molecular mechanisms in this paper, progress has been made in recent years, which has advanced our understanding of tumor angiogenesis to some extent. However, considering the dual complexity of tumor angiogenesis and the potential involvement of various molecular mechanisms, our current understanding of the role of ncRNAs in tumor angiogenesis may only scratch the surface.

During the literature review process, we also focused on exploring the clinical applications of ncRNAs. Unfortunately, there are currently no real applications of ncRNAs in clinical therapy. We speculate on several reasons for this. Firstly, although substantial achievements have been made in understanding molecular mechanisms in humans, there is still a lack of breakthroughs due to similarities or redundancies among many studies. This might be limited by current molecular biology techniques. Secondly, each study highlights its ncRNA as having noteworthy clinical implications for tumor angiogenesis; however, identifying which ones occupy central positions within regulatory networks and serve as viable drug targets likely requires deep collaboration among fields such as artificial intelligence, molecular mechanism research, and drug development to integrate complex regulatory networks. Thirdly, tumor angiogenesis is an ongoing process occurring within a complex tumor microenvironment, which further complicates the application of ncRNAs in clinical settings. Therefore, more advanced models need to be developed for research purposes. Finally, the optimization of drug delivery systems plays a crucial role in reducing patient immune responses and determining the success of ncRNA therapy. Fortunately, the field of nanomaterials has experienced rapid development in recent years. With the continuous development of multiple disciplines, it is foreseeable that there will be more clinical applications based on ncRNA therapy in the future.

## Figures and Tables

**Table 1:** Targets and dysregulation of miRNA associated with tumor angiogenesis.

MiRNA	Target Genes	Cancer Types	The Role in Tumor Angiogenesis	References
miR-130b-3p	HOXA5	Hepatocellular Carcinoma	Promoter	[28]
miR-130b-3p	MBNL1	Head and Neck Squamous Cell Carcinoma	Promoter	[29]
miR-519a-3p	DUSP2	Gastric Cancer	Promoter	[30]
miR-30b-5p	GJA1	Pancreatic Cancer	Promoter	[31]
miR-197-3p	TIMP2/3	Lung Adenocarcinoma Metastasis	Promoter	[32]
miR-543	MTA1	Non-small Cell Lung Cancer	Promoter	[33]
miR-21-5p	PTEN, PDCD4, and RECK	Non-small Cell Lung Cancer	Promoter	[34]
miR-3157-3p	TIMP/KLF2	Non-small Cell Lung Cancer	Promoter	[35]
miR-619-5p	RCAN1.4	Non-small Cell Lung Cancer	Promoter	[36]
miR-181a	RAD17	Esophageal Cancer	Promoter	[37]
miR-205	PTEN	Ovarian Cancer	Promoter	[38]
miR-21-5p	KRIT1	Colorectal Cancer	Promoter	[39]
miR-N-72	CLDN18	Colorectal Cancer	Promoter	[40]
miR-103a-3p	LATS2 and SAV1	Colorectal Cancer	Promoter	[41]
miR-155-5p	SOCS1	Melanoma	Promoter	[42]
miR-145	IRS1	Breast Cancer	Inhibitor	[43]
miR-206	Met	Colorectal Cancer	Inhibitor	[44]
miR-378a-3p	TRAF1	Hepatocellular Carcinoma	Promoter	[45]
miR-375	PDGFC	Hepatocarcinoma	Inhibitor	[46]
miR-877-3p	FGF2	Osteosarcoma	Inhibitor	[47]
miR-CT3	VEGF-A	Osteosarcoma	Inhibitor	[48]
miR-495	ATP7A	Esophageal Cancer	Inhibitor	[49]

**Table 2:** Targets and dysregulation of lncRNA associated with tumor angiogenesis.

Basic Mechanisms	LncRNA	Target Genes	Cancer Types	The Role in Tumor Angiogenesis	References
Sponging miRNAs (lncRNA-miRNA-targeted gene)	LncRNA ZNRD1-AS1	miR-942/TNS1	Lung Cancer	Promoter	[66]
	LINC00173.v1	miR-511-5p/VEGF-A	Lung Squamous cell carcinoma	Promoter	[67]
	LncRNA PCAT6	miR-4723-5p/VEGFR2	TNBC	Promoter	[68]
	LncRNA NR2F1-AS1	miRNA-338-3p/IGF-1	Breast Cancer	Promoter	[69]
	LncRNA NORAD	miR-211-5p/FOXD1	Hepatocellular Carcinoma Cells	Promoter	[70]
	LncRNA MYLK-AS1	miR-424-5p/E2F7	Hepatocellular Carcinoma	Promoter	[71]
	LncRNA miR503HG	miR-15b/PDCD4	Hepatocellular Carcinoma	Inhibitor	[72]
	LncRNA CRART16	miR-122-5p/FOS	Gastric Cancer	Promoter	[73]
	LncRNA NKX2-1-AS1	miR-145-5p/SERPINE1	Gastric Cancer	Promoter	[74]
	LncRNA H19	miR-138/HIF-1α	Glioma	Promoter	[75]
	LncRNA H19	miR-342/Wnt5a	Glioma	Promoter	[76]
	LncRNA DANCR	miR-145/VEGF	Ovarian Cancer	Promoter	[77]
	LncRNA LOC100129620	miR-335-3p/CDK6	Osteosarcoma	Promoter	[78]
	LncRNA HOTAIR	miR-126/EGFL7	Renal Cell Carcinoma	Promoter	[79]
	LncRNA IGKJ2-MALLP2	miR-1911-3p/p21	Laryngeal Squamous Cell Carcinoma	Inhibitor	[80]
Interacting (lncRNA- Proteins)	LncRNA SNHG5	IGF2BP2	Breast Cancer	Promoter	[81]
	LncRNA RAB11B-AS1	RNA Pol II	Breast Cancer	Promoter	[82]
	LncRNA PAARH	HIF-1α	Hepatocellular Carcinoma	Promoter	[83]
	LncRNA MAGI2-AS3	HEY1	Clear Cell Renal Cell Carcinoma	Inhibitor	[84]
	LncRNA RP11-536 K7.3	SOX2	colorectal cancer	Promoter	[85]
	LncRNA HITT	YB-1	Colon Cancer	Inhibitor	[86]

## Data Availability

No additional data.
